# Supplementary material for: Successive Osteosarcoma Relapses after the First Line O2006/Sarcome-09 Trial: What Can We Learn for Further Phase-II Trials?
Source: Cancers (Basel). 2021 Apr 2;13(7):1683. doi: 10.3390/cancers13071683 (PMC8038261; doi:10.3390/cancers13071683)
Supplement: Supplementary file 1 [file cancers-13-01683-s001.pdf]

Successive Osteosarcoma Relapses after the First Line O2006/Sarcome-09 Trial: What Can We Learn for Further Phase-II Trials?

Eric Thebault, Sophie Piperno-Neumann, Diep Tran, Hélène Pacquement, Perrine Marec-Berard, Cyril Lervat, Marie-Pierre Castex, Morgane Cleirec, Emmanuelle Bompas, Jean-Pierre Vannier, Dominique Plantaz, Laure Saumet, Cecile Verite, Olivier Collard, Claire Pluchart, Claire Briandet, Laure Monard, Laurence Brugieres, Marie-Cécile Le Deley and Nathalie Gaspar

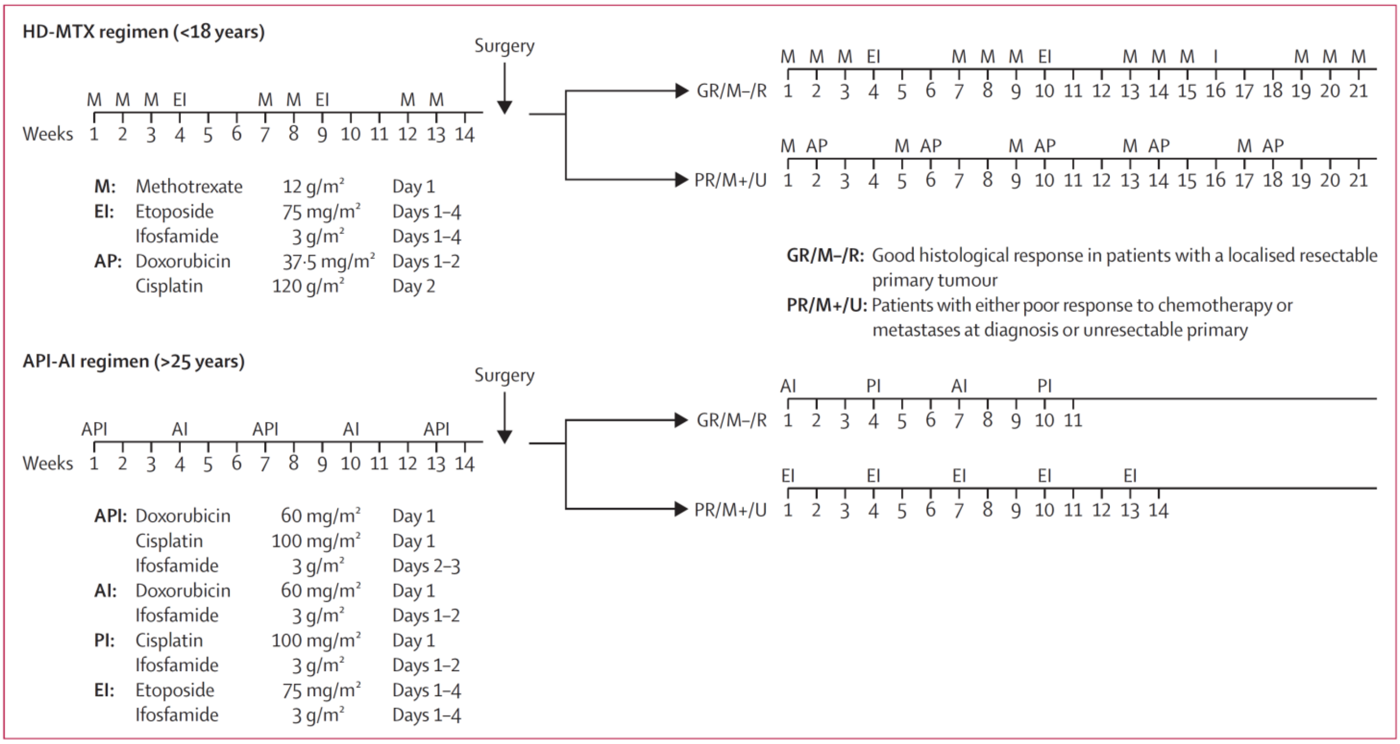

Figure S1. OS2006/Sarcome-09 Backbone chemotherapy scheme.

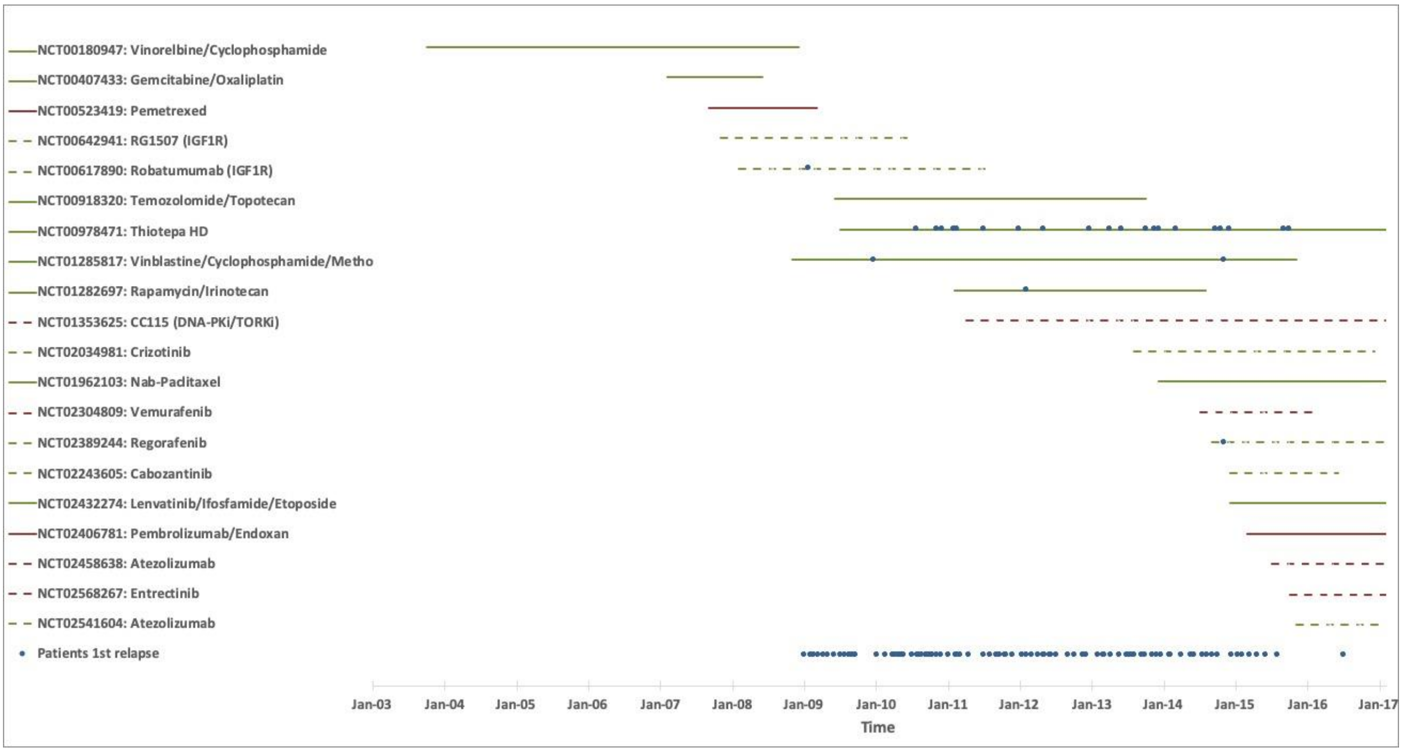

A

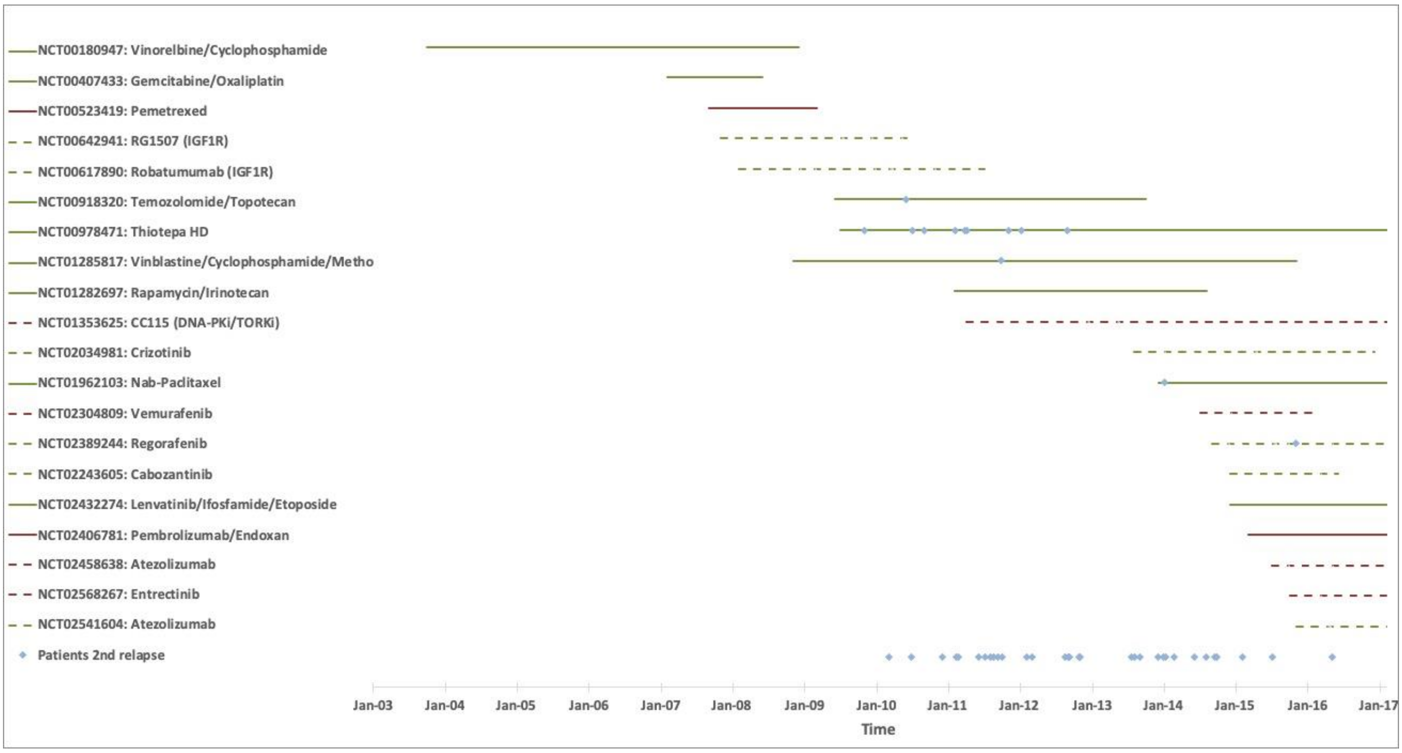

B

Figure S2. Active Phase-I and Phase-II trials for relapsed osteosarcoma in France between March 2007 and June 2016. **A**-Trials open for first osteosarcoma relapses. **B**-Trials open for second and subsequent osteosarcoma relapses. Plain line corresponds to trials with chemotherapy; dot line to trials without any chemotherapy agent; Green line to trials with paediatric population included; and red line to trials without paediatric population included (ie criterion ≥18 years).

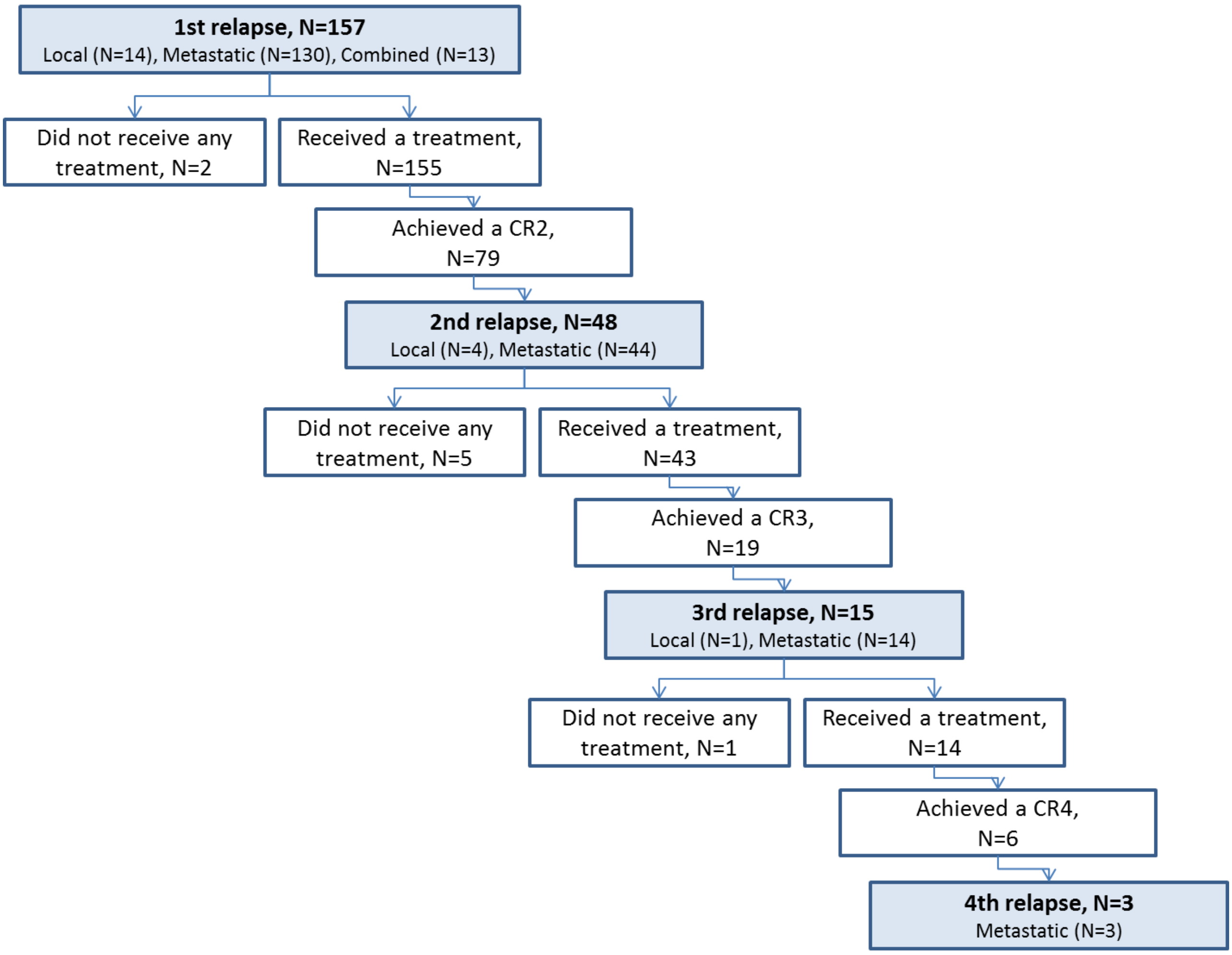

Figure S3. First and subsequent relapses of osteosarcoma.

Table S1. Open Trials for recurrent osteosarcoma in France between 04/2007 and 06/2016 (trials extracted from a previous paper 16)

Publication Reference	Drug Tested	Diseases Included	Inclusion Criteria		Inclusion		Primary Endpoint	Response Evaluation Criteria	Trial	Randomization
			Age	Other	Start	End				
**NCT00180947**	**Vinorelbine + Endoxan**	**Solid tumours**	**12m-25y**	**Relapse without standard therapy**	**oct-03**	dec-08	2 months Response rate	WHO	Phase II	No
**NCT00407433**	Gemcitabine + Oxaliplatin	Solid tumours	6m-21y	Relapsed or refractory tumours	feb-07	jun-08	2 months Response rate	WHO	Phase II	No
**NCT00523419**	Pemetrexed	Osteosarcoma	>18y	Already received first line chemotherapy	sept-07	mar-09	Best 1,5 months response rate	RECIST	Phase II	No
**NCT00642941**	Teprotumumab	Sarcoma	>2y	Recurrent or refractory sarcoma (osteosarcoma or other)	nov-07	jun-10	6 months response rate		Phase II	No
**NCT00617890**	Robatumumab	Osteosarcoma and Ewing's sarcoma	>4y	Relapse sarcoma operable or not	feb-08	aug-11	DCR (CR or PR)	RECIST or WHO	Phase II	No
**NCT00918320**	Temozolomide + Topotecan	Solid tumours	6m – 20y	Relapsed or refractory tumours	jun-09	oct-13	2 months Response rate		Phase II	No
**NCT00978471**	Thiotepa HD	Osteosarcoma	1-50ans	1st relapse or 2nd relapse after surgery only; Surgery of all the sites of the relapse	jul-09	dec-17	2 years OS	0	Phase II	Yes
**NCT01285817**	Celecoxib+Vinblastine + Cyclophosphamide + Methotrexate	Solid tumours	4y-21y	Progression or Relapse; Refractory	nov-10	nov-15	PFS	?	Phase II	No
**NCT01282697**	Rapamycin + Irinotecan	Solid tumours	1y-21y	Relapsed or refractory tumours	feb-11	aug-14	Maximum tolerated dose	0	Phase I	No
**NCT01353625**	CC-115 (mTOR inhibitor)	Solid and Hematologic cancers	>18y	Progression or not tolerated standard therapy; Never treated with mTOR inhibitor	apr-11	sept-18	PD et PK	0	Phase I	No

**NCT02034981**	Crizotinib	Hematologic and solid tumours	>1y	No standard therapy; One proven specific alterations among ALK, MET, RON, and ROS1 genes	aug-13	dec-16	2 months Response rate	RECIST	Phase II	No
**NCT01962103**	Nab-Paclitaxel	Solid Tumours	6m-24y	Solid tumours refractory or on relapse or without standard therapeutic	dec-13	dec-18	Best response rate	RECIST	Phase I/II	No
**NCT02304809**	Vemurafenib	Solid tumours or Hematologic cancers	>18y	Unresectable malignancy resistant or refractory; BRAF V600 mutation	jul-14	feb-16	2 months Response rate	RECIST	Phase II	No
**NCT02389244**	Regorafenib	Ewing Sarcoma, Chondrosarcoma, Osteosarcoma, Chondroma	>10y	Progression disease; Metastatic disease not amenable to surgical resection	sept-14	sept-19	PFS	RECIST	Phase II	Yes
**NCT02243605**	Cabozantinib	Osteosarcoma and Ewing's sarcoma	>12y	Relapsed disease; Progressive disease	dec-14	jun-16	6 months Non progression (CR, PR, SD)	RECIST	Phase II	No
**NCT02432274**	Lenvatinib +/- Cyclophosphamide and Etoposide	Solid tumours	2y-25y	Relapsed or refractory tumour that has progressed	dec-14	jun-18	Objective response rate and 4 months PFS	RECIST	Phase I/II	No
**NCT02406781**	Pembrolizumab + Cyclophosphamide	Sarcoma	>18y	Advanced non resectable or metastatic disease; Progression	mar-15	mar-17	6 months response rate	RECIST	Phase II	No
**NCT02458638**	Atezolizumab	Solid tumours	>18y	Progression disease	jul-15	dec-20	4 months Non-progression rate (CR, PR, SD)	RECIST	Phase II	No

**NCT02568267**	Entrectinib	Solid and hematologic cancers	>18y	NTRK1/2/3, ROS1, or ALK gene rearrangement; CNS involvement	oct-15	oct-17	Best response rate	RECIST	Phase II	No
**NCT02541604**	Atezolizumab	Solid tumours	<30y	Relapsed or refractory	nov-15	janv-22	PFS; Best response rate	RECIST	Phase I/II	No

Table S2. Baseline patient and tumour characteristics and first-line treatment of the 157 patients with a relapse.

Initial Characteristics	Number (%)<br>Median (min-max)
**Age at study entry in the OS2006/Sarcome-09 study, in years**	
Median (range)	15.4 (6.4-50.4)
Less than 12 years, N (%)	34 (21.7%)
12-17 years, N (%)	73 (46.5%)
18-25 years, N (%)	30 (19.1%)
>25 years, N (%)	20 (12.7%)
**Primary tumour site**	
Trunk /head and neck, N (%)	14 (8.9%)
Lower limb, N (%)	117 (74.5%)
Upper limb, N (%)	26 (16.6%)
**Metastasis at diagnosis**	24 (15.3%)
Lung metastasis only, N (%)	19 (12.1%)
Bone metastasis only, N (%)	3 (1.9%)
Combined metastasis (Lung + other site), N (%)	2 (1.3%)
**Type of centre for the first line treatment**	
Medical oncology department, N (%)	52 (33.1%)
Paediatric centre, N (%)	105 (66.9%)
**Chemotherapy as first-line treatment (OS2006 protocol)**	
Methotrexate-etoposide-ifosfamide, N (%)	124 (79.0%)
API-AI, N (%)	33 (21.0%)
**Treatment allocated by randomisation**	
No randomisation, N (%)	58 (36.9%)
Without Zoledronate, N (%)	45 (28.7%)
With Zoledronate, N (%)	54 (34.4%)
**Surgery of the primary tumour**	
Conservative surgery, N (%)	139 (88.5%)
Amputation, N (%)	18 (11.5%)
**Histological response of primitive tumour**	
Good histological response (< 10% viable cells), N (%)	89 (56.7%)
Poor histological response (≥ 10% viable cells), N (%)	68 (43.3%)

Table S3. Characteristics of the 39 patients with a unique lung nodule at first relapse, overall and according to treatment received at relapse.

Patient and Tumour Characteristics	Overall<br>N = 39	Surgery alone<br>N = 21	Systemic Treatment<br>N = 18	*p*-value
**Metastasis at diagnosis**				1.00 *
No Metastasis, N (%)	30 (76.9%)	16 (76.2%)	14 (77.8%)	
Lung metastasis +/- other site, N (%)	9 (23.1%)	5 (23.8%)	4 (22.2%)	
**Histological response of primitive tumour**				1.00 *
Good histological response, N (%)	22 (56.4%)	12 (57.1%)	10 (55.6%)	
Poor histological response, N (%)	17 (43.6%)	9 (42.9%)	8 (44.4%)	
**Type of centre**				0.07 *
Medical oncology department, N (%)	10 (25.6%)	8 (38.1%)	2 (11.1%)	
Paediatric centre, N (%)	29 (74.4%)	13 (61.9%)	16 (88.9%)	
**Age at relapse** (years)				
Median (range)	16.7 (8.4-43.6)	17.4 (9.5-43.6)	15.4 (8.4-25.7)	0.18 **
Less than 12 years, N (%)	5 (12.8%)	2 (9.5%)	3 (16.7%)	
12-17 years, N (%)	20 (51.3%)	9 (42.9%)	11 (61.1%)	
18-25 years, N (%)	11 (28.2%)	7 (33.3%)	4 (22.2%)	

Patient and Tumour Characteristics	Overall N = 39	Surgery alone N = 21	Systemic Treatment N = 18	*p*-value
>25 years, N (%)	3 (7.7%)	3 (14.3%)	0 (0%)	
**Time interval from diagnosis to 1st relapse (years)**				
Median (range)	1.7 (0.9-5.7)	1.9 (0.9-5.7)	1.6 (1.2-3.6)	0.75 **
<1 year, N (%)	1 (2.6%)	1 (4.8%)	0 (0%)	
>1 year, N (%)	39 (97.4%)	20 (95.2%)	18 (100%)	
**Diameter of the nodule**				
Median (range)	15 (3-46)	14 (4-43)	16 (3-46)	0.42 **
<5 mm, N (%)	3 (7.7%)	1 (4.8%)	2 (11.1%)	
5-9mm, N (%)	11 (28.2%)	7 (33.3%)	4 (22.2%)	
>=10mm, N (%)	25 (64.1%)	13 (61.9%)	12 (66.7%)	

* Fisher exact test; ** Wilcoxon test of the comparison of the distribution of the continuous variable.

Table S4. Characteristics of the subsequent relapses.

Patient and Tumour Characteristics	First Relapse N = 157	Second Relapse N = 48	Third Relapse N = 15
**Site of relapse**			
Local relapse, N (%)	14 (8.9%)	4 (8.3%)	1 (6.7%)
Metastases only, N (%)	130 (82.8%)	44 (91.7%)	14 (93.3%)
Combined, N (%)	13 (8.3%)	0	0
**Treatment for relapse**			
None, N (%)	2 (1.3%)	5 (10.4%)	1 (6.7%)
Systemic treatment, N (%)	116 (73.9%)	32 (66.7%)	9 (60%)
Surgery, N (%)	107 (68.2%)	25 (52.1%)	8 (53%)
Radiation therapy, N (%)	17 (10.8%)	8 (16.7%)	1 (6.7%)
Radiofrequency ablation, N (%)	2 (1.3%)	2 (4.2%)	1 (6.7%)
Other, N (%)	1 (1.3%)	0 (0%)	1 (6.7%)
**Complete remission obtained**, N (%)	79/155 (51.0%)	19/43 (44.2%)	6/14 (42.9%)
**Further relapse**	48	15	3
**Progression-Free Survival**			
1-year	41.8%	27.1%	36.6%
